# Developing High-Power-Density Electromagnetic Devices with Nanocrystalline and Amorphous Magnetic Materials

**DOI:** 10.3390/nano13131963

**Published:** 2023-06-28

**Authors:** Youguang Guo, Lin Liu, Wenliang Yin, Haiyan Lu, Gang Lei, Jianguo Zhu

**Affiliations:** 1 Faculty of Engineering and Information Technology, University of Technology Sydney, Ultimo, NSW 2007, Australia; youguang.guo-1@uts.edu.au (Y.G.); haiyan.lu@uts.edu.au (H.L.); gang.lei@uts.edu.au (G.L.); 2 School of Electrical and Information Engineering, The University of Sydney, Camperdown, NSW 2006, Australia; jianguo.zhu@sydney.edu.au

**Keywords:** nanocrystalline, amorphous magnetic materials, electromagnetic device, high power density, high frequency, transformer, electrical machine

## Abstract

With the increasing demand for smaller, lighter, and more affordable electromagnetic devices, there is a growing trend toward developing high-power-density transformers and electrical machines. While increasing the operating frequency is a straightforward approach to achieving high power density, it can lead to significant power loss within a limited volume, resulting in excessive temperature rise and device degradation. Therefore, it is crucial to design high-power-density electromagnetic devices that exhibit low power loss and efficient thermal dissipation to address these challenges. Advanced techniques, such as the utilization of novel and advanced electromagnetic materials, hold great promise for overcoming these issues. Specifically, nanocrystalline and amorphous magnetic materials have emerged as highly effective solutions for reducing power loss and increasing efficiency in electromagnetic devices. This paper aims to provide an overview of the application of nanocrystalline and amorphous magnetic materials in transformers and electrical machines, along with key technologies and the major challenges involved.

## 1. Introduction

Over the past few decades, there has been a growing demand for smaller, lighter, and more cost-effective energy conversion devices, resulting in widespread applications of high-power-density transformers and electrical machines [1,2,3,4,5,6,7,8,9,10]. Increasing the operating frequency is a straightforward approach to achieving high power density, which reduces the required materials like copper conductors or magnetic cores, leading to smaller and lighter devices. However, operating at high frequencies can cause excessive power loss and heat generation, leading to device performance deterioration or even failure. Power loss in an electromagnetic device primarily comprises copper loss, core loss, and mechanical loss, with the copper loss being the dominant component at power frequency. As the operating frequency increases, the core loss quickly rises and may even surpass the copper loss. Consequently, researchers have undertaken extensive studies to reduce core loss in electromagnetic devices, and the utilization of advanced magnetic materials holds great promise as a solution [11,12,13,14,15].

Advanced magnetic materials, particularly nanocrystalline and amorphous magnetic materials, exhibit distinct magnetic properties that allow for efficient magnetic flux concentration and minimize energy losses. As a result, they are ideal for implementation in high-power-density electromagnetic devices [16,17,18,19,20,21]. Additionally, integrating these materials into electromagnetic devices can result in both substantial reductions in core loss and improvements in efficiency.

Nanocrystalline magnetic materials typically consist of nanoscale crystalline grains, although it is important to note that not all nanocrystalline materials include an amorphous matrix. While some nanocrystalline materials do possess an amorphous matrix surrounding the crystalline grains, there are also nanocrystalline materials that exist as pure crystalline structures without any amorphous component. These materials achieve their unique properties and enhanced performance through the refinement of the grain size and the presence of high-density grain boundaries. Amorphous materials are distinguished by their unique atomic arrangement, which lacks the medium- and long-range order typically found in crystalline structures. Instead, these materials exhibit a disordered arrangement of atoms, with short-range order present in localized clusters. However, this short-range order does not exhibit periodic repetition, leading to the absence of long-range order throughout the material. The absence of a crystalline structure contributes to the distinct properties and behaviors observed in amorphous materials, including their notable magnetic and electrical characteristics. Both nanocrystalline and amorphous magnetic materials exhibit high magnetic permeability and electrical resistivity, making them well-suited for efficiently concentrating magnetic flux and minimizing power losses in electromagnetic devices. Consequently, they find extensive applications in various fields, including transformers, inductors, and electrical machines [22,23,24,25,26,27]. Nanocrystalline materials offer advantages such as improved saturation magnetization compared to their bulk counterparts and higher operating temperatures, making them suitable for high-power applications. On the other hand, amorphous materials commonly possess excellent soft magnetic properties and low coercivity, making them highly desirable for applications requiring low core losses and high-frequency operation. The selection between nanocrystalline and amorphous magnetic materials depends on the specific application requirements, including desired magnetic properties, operating conditions, and cost considerations. A careful evaluation of the trade-offs and performance characteristics of each material is necessary to determine the most suitable choice for a particular application.

Thus, this article provides an overview of recent advancements in high-power-density transformers and electrical machines using nanocrystalline and amorphous magnetic materials as core materials. The existing challenges and future prospects are also concluded. The paper is structured as follows: Section 2 discusses the development of various types of transformers using nanocrystalline cores, while Section 3 focuses on amorphous magnetic cores. Section 4 presents the design and analysis of electrical machines using these advanced materials as cores. Finally, Section 5 concludes the paper by highlighting the major challenges and potential research directions for future developments.

## 2. Developing High-Frequency High-Power-Density Transformers with Nanocrystalline Magnetic Materials

### 2.1. Research Status and Routes

Nanocrystalline magnetic materials provide distinct advantages over amorphous magnetic materials in the design of high-performance transformers and inductors. One notable advantage is their higher saturation flux density, which allows for increased magnetic energy storage capacity. Additionally, nanocrystalline materials exhibit lower coercivity, enabling efficient magnetization and demagnetization processes. These combined attributes contribute to enhanced performance and overall efficiency in magnetic devices. Secondly, they exhibit a higher permeability at high frequencies, making them ideal for high-frequency applications such as power electronics, telecommunications, and renewable energy systems. Therefore, the use of nanocrystalline magnetic materials has become increasingly popular in the development of high-frequency high-power-density transformers and inductors.

As early as 1996, the potential of nanocrystalline magnetic materials for transformer construction was explored by Draxler and Styblikova [28]. They compared the performance of different core materials, including nanocrystalline, amorphous, and permalloy materials, and highlighted the benefits of using Vitroperm, a nanocrystalline magnetic material produced by Vacuumschmelze. In 1998, Ferch [29] reported on the use of nanocrystalline SiFe alloy Vitroperm cores in light transformers for switched-mode power supplies operating at a frequency of 10–20 kHz. This resulted in a more compact and lightweight system with significantly higher frequency capabilities.

In 2000, Costa et al. [30] designed a flyback converter transformer with a stress-annealed Finemet core, achieving low transformer loss and low temperature rise. Finemet is a nanocrystalline magnetic material produced by Hitachi Metals, Ltd. that is suitable for pulse applications due to its very low loss density and high operating temperature features. In 2005, Shen et al. [31] designed a high-power-density Finemet core transformer for resonant converter systems. Lin et al. [32] investigated the magnetic flux density distribution in the cross-sectional area of the Finemet ribbon in 2009. In 2010, Shafik et al. [33] designed a transformer for high current and low voltage DC/DC converters using Nanoperm, a nanocrystalline magnetic material produced by Magnetec GmbH, realizing very low core loss. In 2013, Seltzman et al. [34] designed, analyzed, and constructed a resonant transformer for high-power-switch mode power supply using a nanocrystalline iron core, validating the high volt-seconds and low loss at high switching frequencies achievable with nanocrystalline iron cores.

In 2014, Sefa et al. [35] conducted a comparative study on medium-frequency transformers with SiFe material of 0.1 mm thickness and nanocrystalline material of 0.018 mm thickness, demonstrating the superiority of the latter for operating frequencies above 3 kHz. In 2017, they designed a medium-frequency power transformer with a nanocrystalline core, which outperformed the one with SiFe sheets [36]. In 2015, Kauder and Hameyer [37] compared the iron losses of standard silicon steel and nanocrystalline materials for power transformers in a dual active bridge converter, showing that the nanocrystalline material Vitroperm has much higher electric resistivity and lower specific iron loss than SiFe materials such as JFE JNEX900, NO10, H80-23L, H85-27L, and M150-30S. Additionally, in 2015, Jiang et al. [38] evaluated and measured a high-frequency transformer with a nanocrystalline Vitroperm 500F core, which has a relative permeability of about 20,000. The transformer efficiency exceeds 99% when the frequency is below 100 kHz and the magnetic flux density is within 0.66 T. In 2016, Warnakulasuriya et al. [39] developed a nanocrystalline core transformer of 100 kW and 20 kHz for a DC/DC converter. They discussed the advantages and disadvantages of using nanocrystalline cores by comparing them with ferrite cores.

In 2017, Li et al. [40] used the finite element method to calculate the core loss of a high-frequency transformer with a nanocrystalline alloy core while taking into account the effects of magnetic hysteresis. In 2018, Grybos et al. [41] investigated the magnetic properties of nanocrystalline composite cores for use in high-frequency transformers and inductors. The nanocrystalline cores have stable magnetic permeability, low power loss density, and the potential to develop a wide range of magnetic parameters. In the same year, Ruiz-Robles et al. [42] designed and fabricated medium-frequency transformers with nanocrystalline cores for DC-DC dual active bridge-type converters. They tested a 1 kVA and 5 kHz lab prototype, which demonstrated an efficiency of over 99%. They also compared two lab-scale medium-frequency transformer prototypes, rated at 1 kVA, 120 V/240 V, and 1 kHz, with nanocrystalline and silicon steel cores. The experimental results revealed that the nanocrystalline core transformer has greater power density and efficiency [43]. In 2019, Liu et al. [44] optimized the design of a high-frequency transformer with a nanocrystalline alloy core and Litz-wire for minimizing copper loss. Based on finite element analysis, the efficiency of the optimized transformer can reach up to 99.6%.

In 2020, Chen et al. [45] presented a design and prototype of a medium-frequency transformer with a nanocrystalline core rated at 1 kV, 200 kVA, and 10 kHz, achieving a power density of MW/m^3^ and an efficiency of 99.45% at a forced air-cooling temperature of 62 °C. In 2021, Li et al. [46] investigated high-frequency transformers using flexible nanocrystalline flake ribbons, achieving a core loss reduction of over 50% compared to their ferrite core counterparts. In 2022, Luo et al. [47] reported the characterization of nanocrystalline flake ribbons for high-frequency magnetic cores. Compared to the ferrites N87 and N27, the nanocrystalline flake ribbon exhibits lower power loss, higher saturation flux density, and better temperature stability in the frequency range of 85–300 kHz, making it an excellent substitute for ferrite in high power density core components.

### 2.2. Brief Comments

The summarized achievements highlighted the significant advantages of nanocrystalline magnetic materials in the design of high-performance transformers and inductors. These materials have been proven to offer superior properties compared to traditional amorphous and silicon steel materials, making them ideal for high-power-density and high-frequency applications. The studies demonstrated that nanocrystalline magnetic materials, such as Vitroperm, Finemet, and Nanoperm, enable the development of more compact and lightweight systems with increased frequency capabilities. They exhibit higher saturation flux density, lower coercivity, and higher permeability at high frequencies, resulting in improved efficiency and power density. This makes them suitable for various applications, including power electronics, telecommunications, and renewable energy systems.

It is worth noting that the development and application of nanocrystalline magnetic materials are not without challenges. The cost of manufacturing nanocrystalline materials is relatively higher, and the fabrication processes are more complex compared to traditional materials. Additionally, the stability of magnetic properties under different temperatures and external magnetic field conditions requires further research and optimization.

In conclusion, the research and practical applications of nanocrystalline magnetic materials in transformers and inductors have shown promising results. Their unique combination of high performance, compact size, and improved efficiency has opened up new possibilities for the advancement of electromagnetic devices. Continued research and development in this field will contribute to further enhancing the performance and reliability of nanocrystalline materials, driving the progress of modern power systems and electronic technologies.

## 3. Developing High-Frequency High-Power-Density Transformers with Amorphous Magnetic Materials

### 3.1. Research Status and Routes

Amorphous magnetic materials are suitable for transformers with low or medium frequencies due to their higher magnetic permeability at lower frequency ranges. They are also more cost-effective than nanocrystalline magnetic materials, making them accessible for specific applications. Milkovic et al. [48] reported the use of an amorphous core transformer magnetic link in an electronic current transformer as early as 1977, which outperformed the Fe-Si core transformer. In 1984, Yamamoto et al. [49] conducted a design study of amorphous core transformers and found that the core loss of the amorphous core transformer was only about 25% of that of a 5000 kVA silicon steel core transformer. Then, Alexandrov et al. [50] presented the design considerations and economic impact of amorphous ferromagnetic materials for distribution transformers in 1987, showing significant economic savings depending on the transformer design. In 1999, Lee et al. [51] developed a three-phase power transformer with superconducting windings and amorphous cores to reduce both copper and core losses. In 2007, Wang et al. [52] achieved an efficiency of 98.5% for a 630 kVA three-phase transformer with high-temperature superconducting windings and amorphous alloy cores.

**Figure 1:**
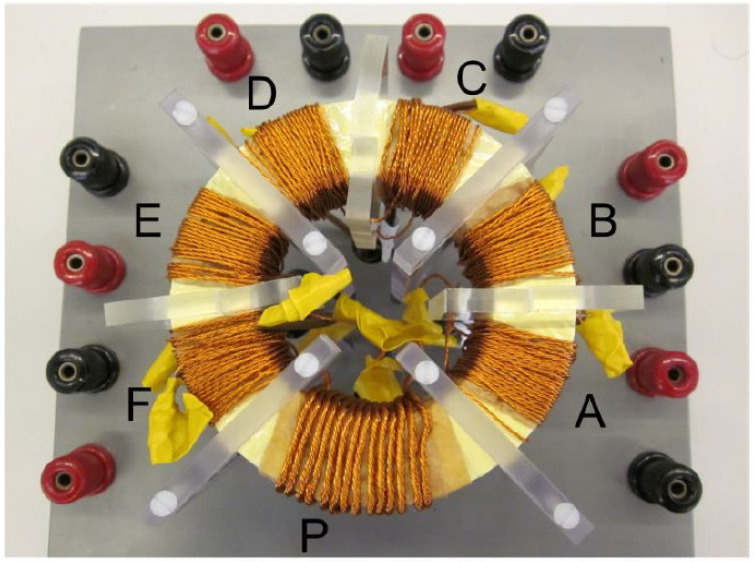
Photos of high-frequency magnetic links with Metglas amorphous alloy cores.

In 2014, Islam et al. [53,54,55,56] designed high-frequency magnetic links for power converters used in grid-connected renewable energy systems with Metglas alloys 2605S3A and 2605SA1 cores, as shown in Figure 1, where the alphabets A, B, C, D, E, and F are the 188 six secondary coils. The design aimed to produce isolated and balanced multiple DC sources for the converter utilizing the magnetic link, while the use of an amorphous core helped reduce the transformer volume and loss. They developed a 1.73 kVA lab-scale system with a modular 5-level cascaded converter to convert 210 V DC to three-phase 1 kV RMS 50 Hz AC.

In 2017, Jafari et al. [57] designed and implemented a multi-winding high-frequency transformer with an amorphous magnetic core to couple multiple converters in a smart microgrid. In 2018, Islam et al. [58,59] developed a common high-frequency magnetic bus with amorphous and nanocrystalline magnetic alloys, such as 2605SA1, 2605S3A, 2705M, 2714A, 2826B, Vitroperm, and Finemet, to replace the common DC bus in order to simplify the integration of multiple sources of existing technologies. In the same year, Xu et al. [60] presented the design of Fe-based amorphous core transformers for pulsed power supplies and switched-mode power supplies operating at medium frequencies. In 2019, Kiran et al. [61] compared the characteristics of high-frequency transformers with amorphous magnetic material Metglas 2605S3A and nanocrystalline material 1k107. Additionally, in 2019, Khan et al. [18] applied an amorphous alloy multi-winding magnetic link of high frequency and high-power-density for generating balanced DC supplies for a five-level neutral point clamped converter. In 2021, Kiran et al. [62] investigated the use of distributed winding topology in amorphous magnetic material-based high-frequency transformers to increase the power transfer capability. In 2022, Liu et al. [63] presented the design and optimization of distribution transformers with amorphous metal cores, highlighting their economic benefits and market competitiveness. Additionally, Zhang et al. [64] studied a shell-type high-power medium frequency transformer with an amorphous magnetic material core for high-power electronic applications.

### 3.2. Brief Comments

Amorphous magnetic materials demonstrate significant advantages and potential in the field of transformers. These materials exhibit higher magnetic permeability at low or medium frequencies, making them an ideal choice for transformers operating in these frequency ranges. Compared to nanocrystalline magnetic materials, amorphous materials offer cost-effectiveness, providing more economically feasible solutions for specific applications.

The research conducted over the past decades showcases the application and performance benefits of amorphous magnetic materials in transformer design. These studies demonstrate that utilizing amorphous magnetic materials can lead to lower core losses, reduced volume, and improved energy efficiency. These factors are crucial for enhancing the performance and efficiency of energy conversion systems, particularly in the realm of renewable energy. For instance, the use of amorphous magnetic materials in the design of high-frequency transformers enables the system to achieve isolated and balanced multiple DC sources while minimizing transformer volume and losses.

However, amorphous magnetic materials may also face challenges in terms of performance degradation at higher frequencies and relatively higher manufacturing costs. Therefore, when selecting transformer materials, it is essential to consider factors such as frequency, cost, and performance holistically. In conclusion, the research and application of amorphous magnetic materials demonstrate their potential and advantages in energy conversion and renewable energy systems. With ongoing technological advancements and improvements, we can expect amorphous magnetic materials to play a more significant role in future transformer designs, making greater contributions to the sustainable development of the energy sector.

## 4. Developing Electrical Machines with Nanocrystalline and Amorphous Magnetic Materials

### 4.1. Research Status and Routes

In addition to their use in transformers, research has also explored the potential applications of nanocrystalline and amorphous magnetic materials in electrical machines. While only a few studies have been conducted on the use of nanocrystalline materials [65,66], a considerable amount of research has been dedicated to the use of amorphous core electrical machines [67,68,69,70,71,72,73,74,75,76,77,78,79,80]. One of the earliest studies was conducted by Mischler et al. [67] in 1981, who constructed and tested an induction motor with an amorphous metal stator. They found that the core loss of the amorphous iron stator was 1 W at 60 Hz, whereas the corresponding core loss of a 0.35 mm M22 silicon iron core was 5 W. In 1982, Johnson et al. [68] presented applications of amorphous metals in electric motors and transformers, reducing core losses by over 70%. They also concluded that the performance is highly dependent on the processing methods of the cores, and significant efforts are required for material handling and accessible processing of the motors and transformers. In 1989, Fukao et al. [69] built and tested a super-high-speed reluctance motor with an amorphous metal Metglas alloy 2505S-2 as both the stator and rotor cores. At 48,000 rpm, the amorphous metal motor had only one-fifth the core loss of the corresponding silicon-iron motor. Furthermore, the cost appears competitive when the system already requires a variable-speed drive. In 1992, Jensen et al. [70] proposed an axial-flux permanent magnet brushless DC motor with a tape-wound amorphous iron Metglas 2605S-2 core, achieving very small core loss.

In 2005, Liew et al. [71] designed an axial-flux permanent magnet motor that used the cut amorphous magnetic material Metglas 2605SA1 as its core. This design offered higher power density and better winding utilization. In 2009, Wang et al. [72] developed an axial gap motor that utilized cut amorphous cores in the stator, which reduced iron loss and saved space. In 2011, Kolano et al. [73] developed a high-speed permanent magnet brushless DC motor with an amorphous magnetic material, Metglas 2605SA1, as the stator core. They found that the iron losses in the amorphous stators were considerably smaller than those in the conventional silicon steel stators. In 2014, Fan et al. [74] reported a motor with an amorphous alloy core that had a higher power density, was 32% smaller in volume, and had a 45% higher power density than the baseline motor. Also in 2014, Dems and Komeza [75] studied the performance characteristics of a high-speed induction motor with an amorphous alloy 2605SA1 core, achieving significantly reduced core losses compared to a conventional 0.5 mm electrical steel core. In 2016, Okamoto et al. [76] applied the amorphous magnetic material 2605HB1M as the core of an interior permanent magnet synchronous motor and achieved a 50% reduction in core loss compared to a non-oriented electrical steel 35H300 core.

In 2020, Li et al. [77] conducted a multi-physics analysis of a yokeless and segmented armature axial-field in-wheel motor utilizing amorphous alloy stator cores, demonstrating its potential as a simple, efficient, and reliable option for electric vehicles. In 2021, Simizu et al. [78] utilized a metal amorphous nanocomposite as the core of a flux-switching permanent magnet motor rated at 2.5 kW and 1400 Hz, indicating its suitability for motor applications with high magnetic switching frequencies. In 2021, Fan et al. [79] developed a high-speed induction motor utilizing amorphous alloy cores, aiming to address power loss and stress issues. In 2022, Chai et al. [80] analyzed the vibration characteristics of a high-speed switched reluctance motor that employed amorphous alloy cores to achieve high efficiency and high power density.

### 4.2. Magnetic Properties and Their Measurements

**Figure 2:**
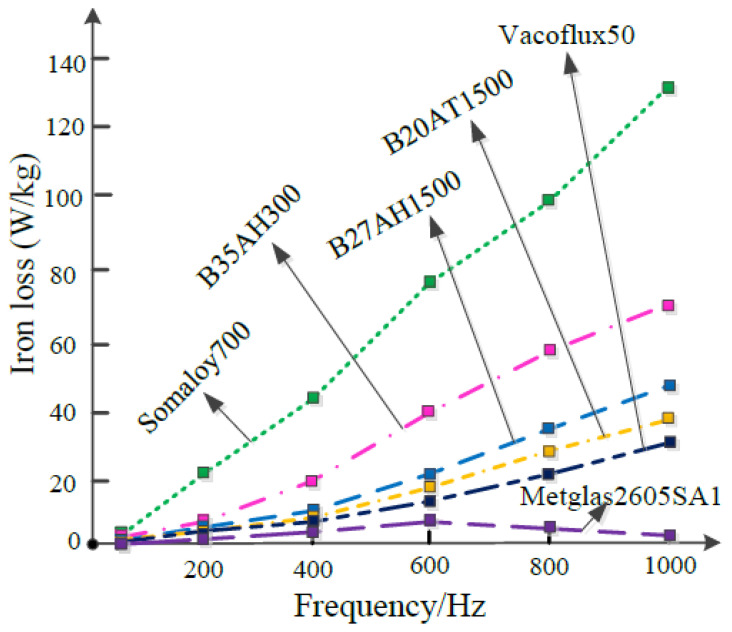
Iron loss of different core materials with respect to frequency [15].

As shown in Figure 2, the iron loss of various motor stator core materials varies with the working frequency. Stators made of amorphous alloy exhibit significantly lower iron losses compared to silicon steel sheets. However, the manufacturing technology for batch production of amorphous alloy stampings is currently limited to simple non-slot structures. Soft Magnetic Composite (SMC) materials can be a suitable alternative for stator cores in high-speed permanent magnet machines. When the motor operates at frequencies higher than 2000 Hz, SMC materials outperform electrical steels in reducing core loss, thereby helping to minimize winding heating.

**Figure 3:**
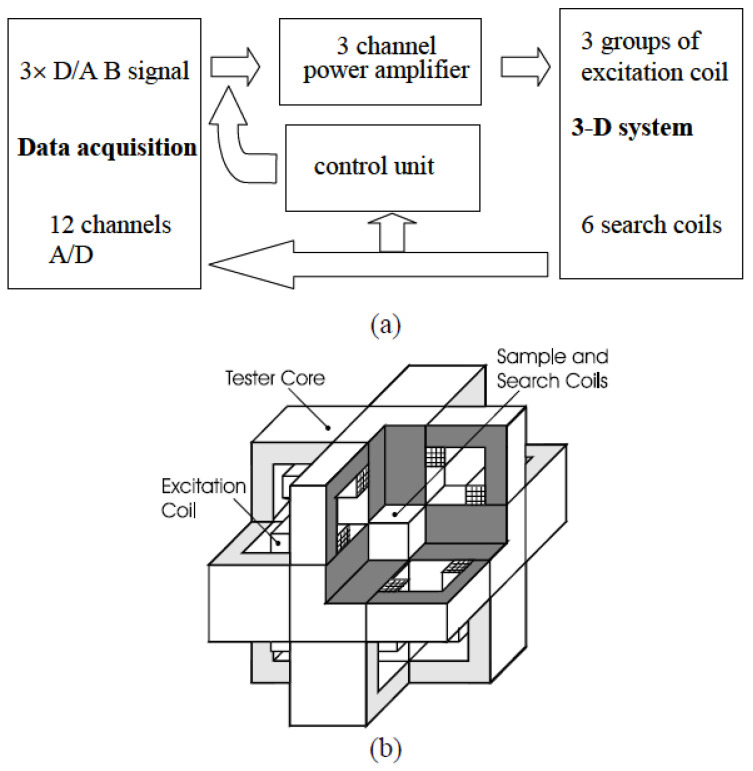
3D Vectorial Magnetic Property measurement system: (**a**) Block diagram and (**b**) structure 330 of 3D view [15].

**Figure 4:**
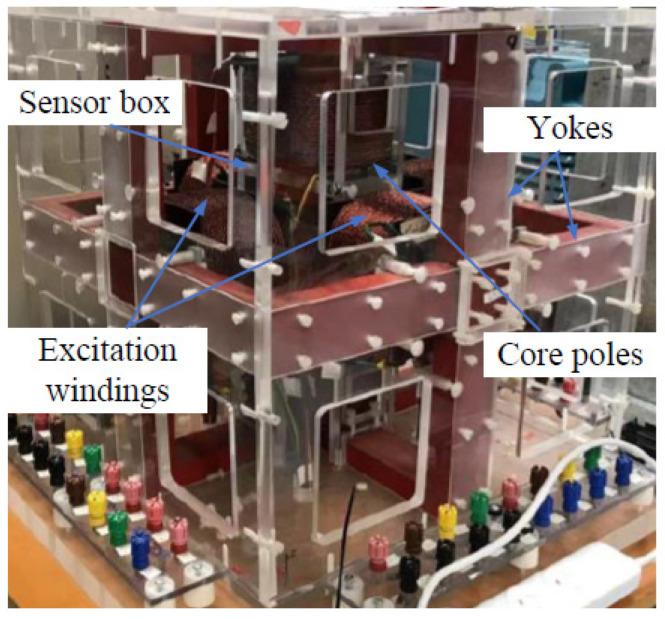
A photo of the 3D vectorial magnetic property measurement system [15].

As for the measurement of 2-D magnetic properties, under the leadership of J. Zhu, the Magnetic Testing Group at the University of Technology Sydney (UTS) developed a square sample single sheet tester [81] for measuring various magnetic materials. This tester is equipped with a computerized digital signal processing system and is capable of measuring **B**-**H** relations and core losses under 1D alternating fluxes in specific directions as well as 2D circular or elliptical rotating fluxes with user-defined axis ratios. In addition, the magnetic field within the core of an electrical machine naturally exists in three dimensions, meaning that the **B** and **H** vectors form irregular loops in 3D space. This characteristic is particularly evident in 3D magnetic flux electrical machines such as claw poles and transverse flux machines [82]. Even with 1D alternating or 2D rotating magnetization, the magnetic material can exhibit 3D magnetic properties due to the motion of magnetic domain walls and magnetization rotation [83]. This phenomenon has been observed in SMC (Soft Magnetic Composite) materials, but it may also occur in amorphous materials and warrants further investigation. In 2001, the authors’ group also developed the world’s first 3D magnetic testing system, as depicted in Figure 3 and Figure 4. The measurement system comprises a 3D magnetic property tester, a computer data acquisition and control system, and a 3-channel power amplifier. The tester consists of six yokes, and three pairs of excitation windings are wound around these yokes. These excitation windings generate 3D magnetic flux in the material sample placed at the center of the tester.

By controlling the magnitudes and phase angles of the excitation currents in the three axes, the tester can produce various flux patterns. These patterns include 1D alternating flux in any specified orientation, 2D circularly or elliptically rotating flux in a plane tilted at a specified angle from an axis, and 3D rotating flux patterns with the loci of the B vector tip forming a specified surface. The selection of the flux pattern depends on the specific measurement requirements. The computer data acquisition and control system, along with the 3-channel power amplifier, facilitate the control and monitoring of the excitation currents as well as the acquisition and analysis of the magnetic field data during the measurement process.

With the utilization of the 3D magnetic testing system, extensive experimental results have been acquired by subjecting various material samples to different magnetization patterns, including 1D alternating flux, 2D circularly rotating flux, and 3D spherical flux densities. More details concerning the experimental results can be found in our previous publications [15,84].

### 4.3. Brief Comments

The research status indicates a growing interest in the application of nanocrystalline and amorphous magnetic materials in electrical machines, expanding beyond their traditional use in transformers. While nanocrystalline materials have received relatively less attention, significant research efforts have been dedicated to exploring the potential of amorphous core electrical machines.

Early studies demonstrated the advantages of using amorphous metal stators, such as reduced core losses compared to traditional silicon iron cores. This finding has prompted further investigation into the performance of amorphous materials in electric motors and transformers. Researchers have explored various motor designs utilizing amorphous magnetic materials, such as axial-flux permanent magnet motors, high-speed brushless DC motors, and reluctance motors. These studies consistently report benefits such as reduced core losses, improved power density, and increased efficiency compared to their conventional counterparts. The use of cut amorphous magnetic materials, such as Metglas alloys, as motor cores has also shown promising results in terms of higher power density, improved winding utilization, and smaller motor size. These advancements have the potential to enhance the overall performance and compactness of electrical machines. Furthermore, the research highlights the potential cost competitiveness of amorphous-core electrical machines, particularly when variable-speed drives are already required in the system. Recent studies have explored the use of amorphous alloy stator cores in electric vehicles, indicating their potential for simple, efficient, and reliable motor designs. Additionally, the application of metal amorphous nanocomposites in flux-switching permanent magnet motors has also demonstrated their suitability for high magnetic switching frequency applications.

Overall, existing achievements have signified the increasing importance of nanocrystalline and amorphous magnetic materials in advancing electrical machine technology. These materials offer significant advantages in terms of improved efficiency, reduced core losses, higher power density, and potential cost competitiveness, making them a promising area for further exploration and development in the field of electrical machines.

## 5. Conclusions and Discussions

The exceptional low-loss characteristics of nanocrystalline and amorphous magnetic materials have made them increasingly popular in electromagnetic device applications. Despite the promising results from the aforementioned studies, there are still several challenging issues that require attention in future research.

Firstly, the magnetic field in a rotating electrical machine and the T-joint of a three-phase transformer are essentially rotational [85,86,87,88]. The magnetic flux density (B) and magnetic field strength (H) vectors are not aligned in the same direction, resulting in two-dimensional (2-D) vectorial magnetization. It has been discovered that the magnetic properties of the materials behave very differently under 2-D vectorial magnetic fluxes than under the traditional one-dimensional (1-D) alternating magnetization. Therefore, it is essential to investigate the magnetic properties of nanocrystalline and amorphous magnetic materials under 2-D magnetizations, although only a few studies have been reported so far [81,89,90,91]. Furthermore, some electrical machines may have three-dimensional (3-D) rotating magnetic fields, and the magnetic properties of the core materials under 3-D magnetizations must also be explored [82,83,84].

Secondly, the appropriate mathematical modeling of magnetic properties is crucial to taking advantage of advanced materials [92,93,94]. The mathematical models should be constructed based on a thorough understanding of the magnetization mechanism, which is dependent on the analysis of a large amount of data obtained under various magnetization conditions from different nanocrystalline and amorphous material samples. Additionally, the magnetic properties may be influenced by various factors such as temperature and stress, and dynamic models may be required to consider the mutual effects of these key factors during the operation process [95,96,97,98].

Lastly, it has been found that direct substitution of conventional silicon steels with advanced materials such as nanocrystalline and amorphous magnetic materials does not necessarily improve performance, and a system-level optimization involving multi-objectives, multi-domains, and multi-physics should be conducted to achieve optimal system-level performance [99,100,101].

## Data Availability

Not applicable.
